# Krill oil supplementation reduces the growth of CT-26 orthotopic tumours in Balb/c mice

**DOI:** 10.1186/s12906-022-03521-4

**Published:** 2022-02-04

**Authors:** Abilasha Gayani Jayathilake, Elif Kadife, Nyanbol Kuol, Rodney Brain Luwor, Kulmira Nurgali, Xiao Qun Su

**Affiliations:** 1 Institute for Health and Sport, Victoria University, Melbourne, 8001 Australia; 2 Department of Surgery, The Royal Melbourne Hospital, The University of Melbourne, Parkville, Australia; 3 Department of Medicine, Western Health, The University of Melbourne, Melbourne, Australia; 4 Regenerative Medicine and Stem Cells Program, Australian Institute for Musculoskeletal Sciences (AIMSS), Melbourne, Australia

**Keywords:** Krill oil supplementation, Colorectal cancer, CRC tumour growth, Ki-67, CD-31, Caspase-7, PARP, EGFR, ERK 1/2, AKT

## Abstract

**Background:**

We have previously reported that the free fatty acid extract (FFAE) of krill oil (KO) significantly inhibits the proliferation and migration, and induces apoptosis of colorectal cancer (CRC) cells. This study aimed to investigate the in vivo efficacy of various doses of KO supplementation on the inhibition of CRC tumour growth, molecular markers of proliferation, angiogenesis, apoptosis, the epidermal growth factor receptor (EGFR) and its downstream molecular signalling.

**Methods:**

Male Balb/c mice were randomly divided into four groups with five in each group. The control (untreated) group received standard chow diet; and other three groups received KO supplementation at 5%, 10%, and 15% of their daily dietary intake respectively for three weeks before and after the orthotopic implantation of CT-26 CRC cells in their caecum. The expression of cell proliferation marker Ki-67 and angiogenesis marker CD-31 were assessed by immunohistochemistry. The expression of EGFR, phosphorylated EGFR (pEGFR), protein kinase B (AKT), pAKT, extracellular signal-regulated kinase (ERK1/2), pERK1/2, cleaved caspase-7, cleaved poly (ADP-ribose) polymerase (PARP), and DNA/RNA damage were determined by western blot.

**Results:**

KO supplementation reduced the CRC tumour growth in a dose-dependent manner*;* with 15% of KO being the most effective in reduction of tumour weight and volume (68.5% and 68.3% respectively, *P* < 0.001), inhibition of cell proliferation by 69.9% (*P* < 0.001) and microvessel density by 72.7% (*P* < 0.001). The suppressive effects of KO on EGFR and its downstream signalling, ERK1/2 and AKT, were consistent with our previous in vitro observations. Furthermore, KO exhibited pro-apoptotic effects on tumour cells as indicated by an increase in the expression of cleaved PARP by 3.9-fold and caspase-7 by 8.9-fold.

**Conclusions:**

This study has demonstrated that KO supplementation reduces CRC tumour growth by inhibiting cancer cell proliferation and blood vessel formation and inducing apoptosis of tumour cells. These anti-cancer effects are associated with the downregulation of the EGFR signalling pathway and activation of caspase-7, PARP cleavage, and DNA/RNA damage.

**Supplementary Information:**

The online version contains supplementary material available at 10.1186/s12906-022-03521-4.

## Background

Colorectal cancer (CRC) is the third most common cancer and the second highest cause of cancer-related deaths in the world [1]. CRC initiates from a gradual progression of genetic and epigenetic alterations in the colonic epithelium. The genetic alterations associated with CRC are the mutation in the *adenomatous polyposis coli* (*APC*) gene and inactivation of the tumour suppressor genes, *TP53* (17p) and *DCC* (18q). Epigenetic alterations including DNA methylation, histone marks, chromatin remodelling and noncoding RNAs (ncRNAs) also play pivotal roles in the development of CRC [2–5]. Genetic and epigenetic aberrations are involved in a complex network and can both predispose to or cause the development of each other [6]. Therefore, understanding epigenetic regulation of gene and/or protein expression can help to develop novel chemotherapy for the treatment of CRC. Multiple factors have been found to be associated with epigenetic regulation and one of such factors is EFGR [7].

It is difficult to detect CRC at the initial stages. The diagnosis is often made at the advanced stages when the tumour has metastasised to other parts of the body. The most common metastasis sites for CRC are the lymph nodes, liver, lungs, peritoneum and the nervous system [8, 9]. Tumour angiogenesis, resistance to apoptosis and overexpression of EGFR signalling pathways have been identified as factors contributing to tumour growth and metastasis [10–12]. These events, together with increased cell proliferation correlate with the prognosis of CRC [13, 14]. Thus, the development of an effective therapeutic/adjunct agent that is capable of overcoming these events would be useful to control the CRC growth, given that the currently available chemotherapies are associated with many adverse side effects.

Over the past ten years, several studies have shown that krill oil has therapeutic potential in the treatment of chronic disorders, including inflammation, cardiovascular disease, hyperlipidemia, arthritis, neurological disorders, kidney disease and diabetes [15–17]. Krill oil extracted from *Euphausia surperba*, a crustacean species found in the Southern Ocean, has been used as an alternative to fish oil due to its high level of long-chain omega-3 polyunsaturated fatty acids (LC n-3 PUFA) [16, 17]. Furthermore, it has also been reported that the presence of astaxanthin in krill oil increases the stability of LC n-3 PUFA and protects them from oxidation that may lead to better health outcomes than fish oil [18].

Our recent studies showed that the free fatty acid extract (FFAE) of krill oil suppresses the proliferation and induces the apoptosis of human CRC cells through the activation of caspases 3 and 9 and possibly via the mitochondrial death pathway [19, 20]. Zhu et al. [21] and Su et al. [22] also reported the anti-proliferative effect of krill oil on CRC and osteosarcoma cells. Recently, Zheng and co-authors have observed that krill oil treatment can effectively reduce the growth of various cancer cells such as breast, leukaemia, hepatocellular carcinoma, prostate cancer attributed to the E-configuration structures of krill oil’s LC n-3 PUFA, eicosapentaenoic acid (EPA) and docosahexaenoic acid (DHA) [23]. Up to now, only one animal study compared the effect of krill oil treatment with eicosapentaenoic acid monoglyceride (MAG-EPA) and EPA ethyl ester (EPA EE) on colorectal tumour growth in nude mice [24]. It was found that krill oil treatment slightly reduced tumour latency and growth compared to control animals.

In this study, we investigated the effects of various doses of krill oil supplementation on CRC tumour growth in orthotopically implanted CT-26 cells in Balb/c mice. The impact of krill oil supplementation on tumour proliferation and angiogenesis was evaluated through the analysis of Ki-67 and CD-31, respectively. Furthermore, the modulatory role of krill oil in vivo on the epidermal growth factor receptor (EGFR) and its downstream molecular signalling, extracellular signal-regulated kinase 1/2 (ERK1/2), and protein kinase B (AKT) were determined. Moreover, the effects of krill oil supplementation on tumour cell apoptosis were determined through the analysis of expression of caspase-7, cleaved poly (ADP-ribose) polymerase (PARP), and DNA/RNA damage.

## Methods

### CT-26 cells and culture conditions

The mouse colon cancer cell line, CT-26, was obtained from the American Tissue Culture Collection (ATCC), Manassas, VA, USA (Catalogue No. CRL-2638). Cells were cultured in RPMI1640 medium (Sigma Aldrich, Castle Hill, NSW, Australia) supplemented with foetal calf serum (FCS, 10%) (Hyclone Quantum Scientific, Clayton South, VIC, Australia), glutamine (10 mM), 4–2-hydroxyethyl-1-piperazineethanesulfonic acid, sodium pyruvate (10 mM), and penicillin (100U/mL)/streptomycin (100 µg/mL) (Sigma Aldrich, Castle Hill, NSW, Australia) at 37 °C in 5% CO_2_ humidified atmosphere. The trypan blue exclusion assay was used to assess the viability of the cancer cells. More than 90% of viable cells obtained after trypsinization of the monolayer culture were used for CRC induction.

### Experimental procedure

**Fig. 1:**
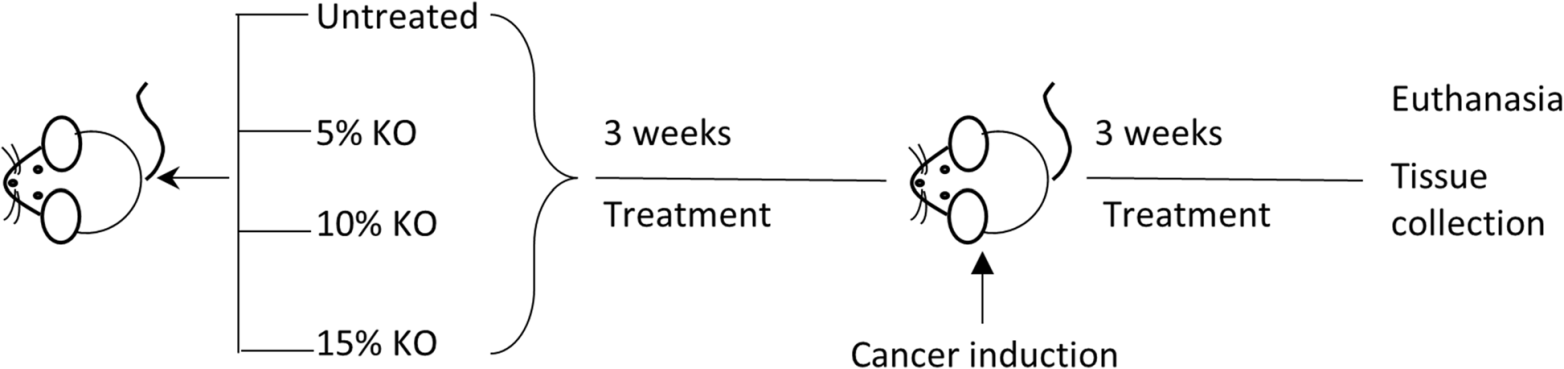
The schematic overview of the study design investigating the effects of different doses of krill oil supplementation in a mouse model of colorectal cancer

The animal study was approved by the Victoria University Animal Ethics Committee (AEC No. 17/008). The study is reported in accordance with ARRIVE guidelines. The mice were maintained in accordance with the guidelines of the Australian National Health and Medical Research Council Code of Conduct on the care and use of laboratory animals for scientific purposes. Seven-week-old male Balb/c mice (*n* = 20, weighted 18–25 g) were obtained from the Animal Resources Centre (Perth, Australia). Animals were housed in OptiMouse cages under pathogen-free conditions with a 12-h light/dark cycle in a well-ventilated room at 22 °C with free access to food and water. Immediately after the arrival, animals were randomised into four groups (untreated group and three treatment groups) with *n* = 5 in each group followed by a five-day acclimatisation period. At the end of acclimatisation, Group 1 (untreated/control) continued their standard chow diet (manufactured by Specialty Feeds, Western Australia); Group 2 received a standard chow diet supplemented with 5% krill oil; Group 3 was given standard chow diet supplemented with 10% krill oil, and Group 4 received standard chow diet supplemented with 15% krill oil as shown in Fig. 1. Krill oil, a product from Swisse Wellness Pty Ltd., Victoria, Australia was purchased from a local pharmacy. It contained 13% EPA, 7.5% DHA and 0.03% Astaxanthin. The amount of krill oil provided to individual animals was calculated based on their feed consumption rate (g/day) and adjusted regularly. Oil supplement was mixed with the standard rodent diet by loading into a pre-calibrated hole in the pellet. Diets supplemented with krill oil were prepared fresh and replaced daily to prevent the oxidation of n-3 polyunsaturated fatty acids. The dietary supplementation regime followed the study by Bathen et al. [25], with three weeks before and after cancer cell implementation to the animals. At the end of three weeks of pre-treatment, CT-26 cells (1 × 10^6^ cells in 25 µL Matrigel, Sigma, Australia) were injected into the caecum of the mice to induce cancer as described before [26]. After a 3-day recovery period, all animals continued their respective dietary treatment for another three weeks. During the study period, food intake was monitored every day and the body weight was measured twice a week. After 21 days of CT-26 cell inoculation or when the tumour size was over 1 cm^3^, animals were culled by overdosing with pentobarbitone (100 mg/kg) [27]. Kaplan–Meier survival analysis was used to assess the survival rate of animals in each group [28]. The tumours were immediately removed, and their weight and volume were measured. Tumour tissues were snap-frozen in liquid nitrogen and stored at -80 °C for later western blotting or fixed with Zamboni’s fixative (2% formaldehyde containing 0.2% picric acid) overnight at 4 °C followed by 3 × 10 min washes with dimethyl sulfoxide (DMSO) (Sigma, Australia) and 3 × 10 min washes with phosphate-buffered saline (PBS). Tissues were frozen in moulds containing 100% Optimum Cutting Temperature compound (OCT Sakura, Tissue Tek, USA) in liquid nitrogen-cooled isopentane, and stored at -80 °C until used for immunohistochemistry analysis.

### Immunohistochemistry for Ki-67 and CD-31

OCT-embedded tumour sections were cut using a cryostat (Leica CM 1950 Biosystems, Germany) at a thickness of 10 µm. Endogenous peroxidase activity was blocked by incubating slides with 10% donkey serum at room temperature for 1 h. After 2 × 10 min washes with PBS, tumour sections were incubated with primary anti-Ki-67 rabbit monoclonal antibody (1:500 dilution, Abcam, United Kingdom) and anti-CD-31 rat monoclonal antibody (1:500 dilution, Abcam, USA) at 4 °C overnight. Following 3 × 10 min washes with PBS-T (Tween-20 0.05%), tumour sections were incubated with the secondary antibodies, donkey anti-rabbit Alexa Fluor 488 and donkey anti-rat Alexa Fluor 594 (1:250, Jackson ImmunoResearch Laboratories, USA) at room temperature for 2 h. Slides were then washed 3 × 10 min with PBS-T before incubation with 4’,6-diamidine-2’-phenylindole dihydrochloride (DAPI, 14 nM, Life Technologies, Australia) for 1 min. Finally, slides were washed with PBS-T for 10 min and cover-slipped using a fluorescent mounting medium (DAKO, Australia). Eight images of each sample at 40 × magnification were taken with the Eclipse Ti confocal laser scanning system (Nikon, Japan). The excitation wavelengths for Alexa Fluor 488 and Alexa Fluor 594 were adjusted to 488 nm and 559 nm respectively. All images were then calibrated to standardize for a minimum basal fluorescence and converted to binary. Fluorescence intensity was measured using Image J software (National Institute of Health, USA). All slides were coded, and analysis was performed blindly.

### Western blot

The expressions of pEGFR/EGFR, pERK/ERK ½, pAKT/AKT, active caspase-7, cleaved PARP and DNA/RNA damage proteins were investigated in tumour samples. Frozen tumour samples were homogenised with a polytron homogenizer (Kinematica AG, Switzerland) for 15 s in ice-cold radioimmunoprecipitation assay (RIPA) buffer (pH 7.4, 150 mM NaCl, 0.1% SDS, 0.5% sodium deoxycholate, 1% NP-40 in PBS, Sigma, Australia) containing a protease and phosphatase inhibitors cocktail (Roche Applied Science, USA). The lysis was centrifuged at 12,000 rpm for 20 min at 4 °C and supernatants were used for western blot analysis. Pierce bicinchoninic acid (BCA) assay (Thermo Fisher Scientific, Australia) was performed to determine the protein concentration. An equal amount of protein sample (12 µg/lane) was loaded onto Mini-PROTEAN® TGXTM (4–20%) stain-free precast gel (Bio-Rad, USA) and separated by 10% sodium dodecyl sulfate–polyacrylamide (SDS-PAGE) gel electrophoresis, then the membrane was blocked with 5% skim milk in PBST (0.1% Tween-20) by incubating at room temperature in a 40 rpm speed shaker for 90 min. The membrane was allowed to react with primary antibodies against pEGFR (1:1000, rabbit, Tyr 1068), EGFR (1:1000, rabbit, monoclonal antibody (mAb), D38B1), pERK 1/2 (1:1000, rabbit, mAB, 9101), ERK 1/2 (1:1000, rabbit, H72), pAKT (1:1000, rabbit, mAB, 9271), AKT antibody (1:1000, rabbit, 9272) mAB), caspase-7 (1:1000, rabbit, Asp198), PARP (1:1000, rabbit, 9542), (all from Cell Signaling Technology, USA), DNA/RNA damage (1:500, mouse, anti-8-OHdG mAB, ab62623 [15A3], Abcam, USA), and glyceraldehyde-3-phosphate dehydrogenase (GAPDH) (1:2000 dilution, rabbit, Santa Cruz Biotechnology, USA) used as the loading control and incubated overnight at 4 °C. Following 3 × washes in PBS-T (0.1% Tween-20) the membrane was incubated with secondary antibodies, goat anti-rabbit IgG H&L horseradish peroxidase (HRP) (Abcam, ab6721, USA) and horse anti-mouse IgG (1:10,000, Cell Signaling Technology, MA, USA) at room temperature for 1 h. Again, the membrane was washed three times in PBS-T (0.1% Tween-20). The protein detection was carried out using enhancing chemiluminescence reagents (Clarity™ Western ECL Substrate, Bio-Rad, USA). Chemiluminescence signals were captured using the FUSION FX System (USA). The expression level of each protein was quantified using Fusion Capt Advance FX7 software. The results were verified through at least three individual experiments.

### Statistical analysis

All data were analysed using SPSS 22 software (IBM, USA). Mixed model ANOVA was used to determine the significance between treatments. The significance of repeated measures at different time points was analysed using one-way ANOVA. Post-hoc analysis was conducted using the Tukey HSD test for multiple comparisons. *P* < 0.05 was considered significant. The Kaplan–Meier survival analysis was performed to assess the survival rate of animals in each group using the formula: Survival Rate = (number of subjects living at the start – number of subjects died)/ number of subjects living at the start. One-way ANOVA was performed to compare the survival rate of animals in different experimental groups. The results were expressed as mean ± SD in texts and mean ± SEM in figures.

## Results

### Effects of different doses of krill oil supplementation on animal survival rate and tumour growth

**Fig. 2:**
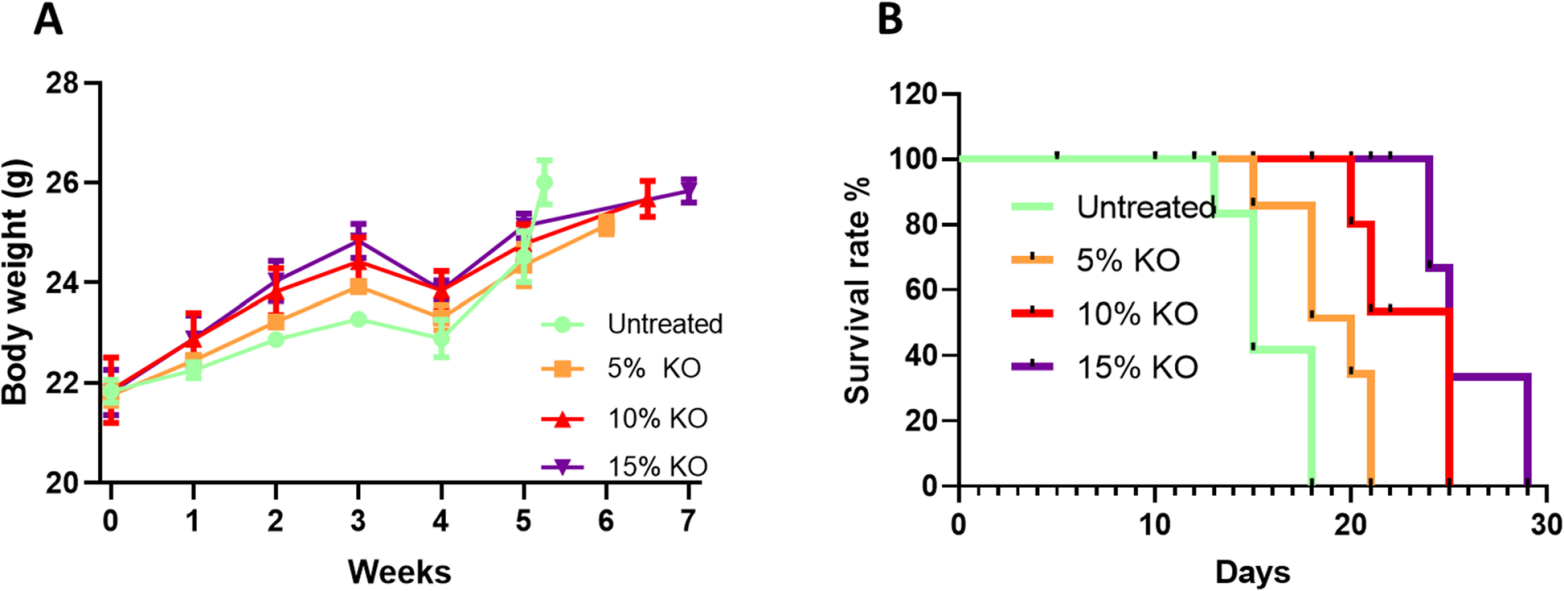
Effects of krill oil supplementation on body weight and animal survival. **A** Changes in the mouse body weight following krill oil treatment compared to the untreated group. **B** Kaplan–Meier survival plot representing the percentage of survival of mice treated with krill oil compared to the untreated group

Animals received a 3-week dietary supplementation of different doses of krill oil in both pre- and post-cancer induction periods (Fig. 1). There were no significant changes in the body weights following krill oil treatment compared to the untreated group (Fig. 2A). The survival rate after the induction of CRC was improved significantly in animals received 10% and 15% of krill oil supplementation compared to the untreated animals, as shown in Fig. 2B.

**Fig. 3:**
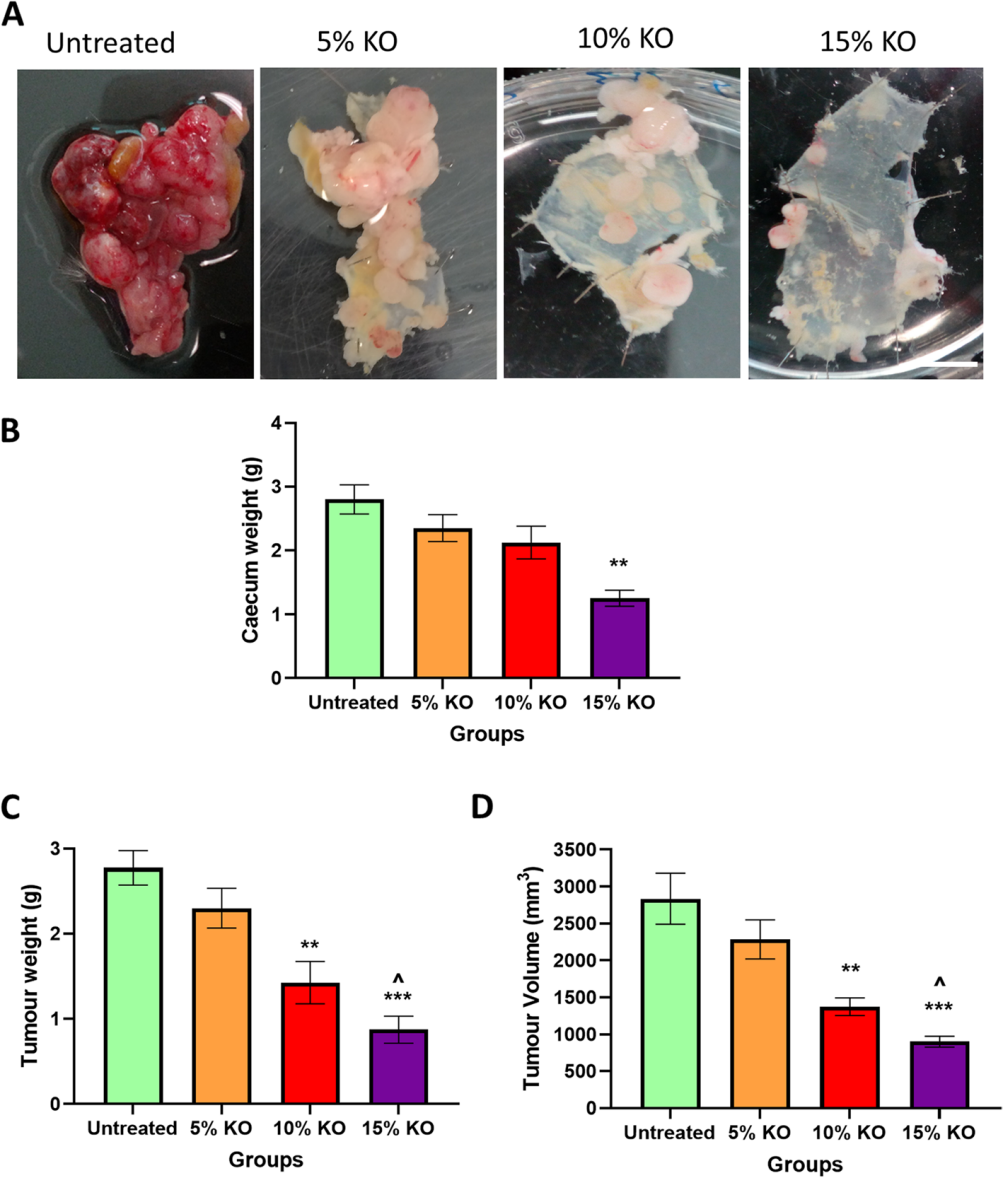
Effects of krill oil supplementation on the caecum and tumour weight and volume in Balb/c mice. **A** Images of mouse caecum implanted with CT-26 cells following treatments with different doses of krill oil compared to the untreated group. **B** The average weight of the caecum with tumours from animals treated with krill oil compared to untreated animals. **C** The mean tumour weight in mice following treatment with different doses of krill oil compared to the untreated group. **D** The mean tumour volume in mice treated with different doses of krill oil compared to untreated animals. *N* = 5 mice per group. The results are expressed as mean ± SEM, *P < 0.05, **P < 0.01, and ***P < 0.001 compared to the untreated group; ^*P* < 0.05 compared to the 5% krill oil-treated group

The total weight of the caecum with tumours excised from animals treated with different doses of krill oil compared to the caecum from the untreated group is presented in Fig. 3A. The average caecum weight of the untreated animals was 2.8 ± 0.2 g, while for the groups fed with 5% and 10% krill oil, caecum weights were 2.4 ± 0.1 g and 2.1 ± 0.2 g, respectively. The reduction was 16.1% and 24.1%, respectively, although not statistically significant. Animals treated with 15% of krill oil showed the lowest caecum weight with an average of 1.5 ± 0.1 g, indicating a significant reduction by 55.4% compared to the untreated group (*P* < 0.01) as shown in Fig. 3B. Moreover, untreated animals fed with a standard chow diet exhibited an aggressive tumour growth in the wall of the caecum, and tumour metastases were observed in various organs/tissues including the colon, small intestine, liver, and diaphragm. Mice fed with 5% of krill oil supplementation also showed more intensive tumour growth in the caecum and metastases in other sites similar to untreated animals. In contrast, all animals treated with 10% or 15% of krill oil had a relatively reduced number and size of tumours in the wall of the caecum compared to the untreated animals, with the most remarkable changes in the group treated with 15% of krill oil. In both 10% and 15% of krill oil-treated groups, only 1 out of 5 mice had metastases to other organs.

The total mean weight and volume of tumours from the untreated group were 2.8 ± 0.3 g and 2659.7 mm^3^, respectively. No differences in the tumour weight and volume were found between the group treated with 5% of krill oil (2.3 ± 0.2 g and 2283, 1 mm^3^, respectively) and the untreated group (Figs. 3C and D). The average tumour weight in these animals was reduced by 17.4% and the volume by 19.4% compared to the untreated animals, although the difference was not statistically significant (*P* > 0.05). For mice fed with the diet supplemented with 10% of krill oil, the average tumour weight and volume were 1.4 ± 0.2 g and 1371.6 mm^3^, respectively. These indicate a reduction in tumour weight by 48.6% and tumour volume by 51.6% compared to the untreated group (*P* < 0.01 for both). The average weight and volume of tumours in animals fed the diet supplemented with 15% of krill oil were 0.9 ± 0.1 g and 898.7 mm^3^, respectively, indicating a significant reduction in tumour weight by 68.5% and volume by 68.3% compared to untreated animals (*P* < 0.001 for both) (Figs. 3C and D).

### Effects of different doses of krill oil supplementation on tumour cell proliferation

**Fig. 4:**
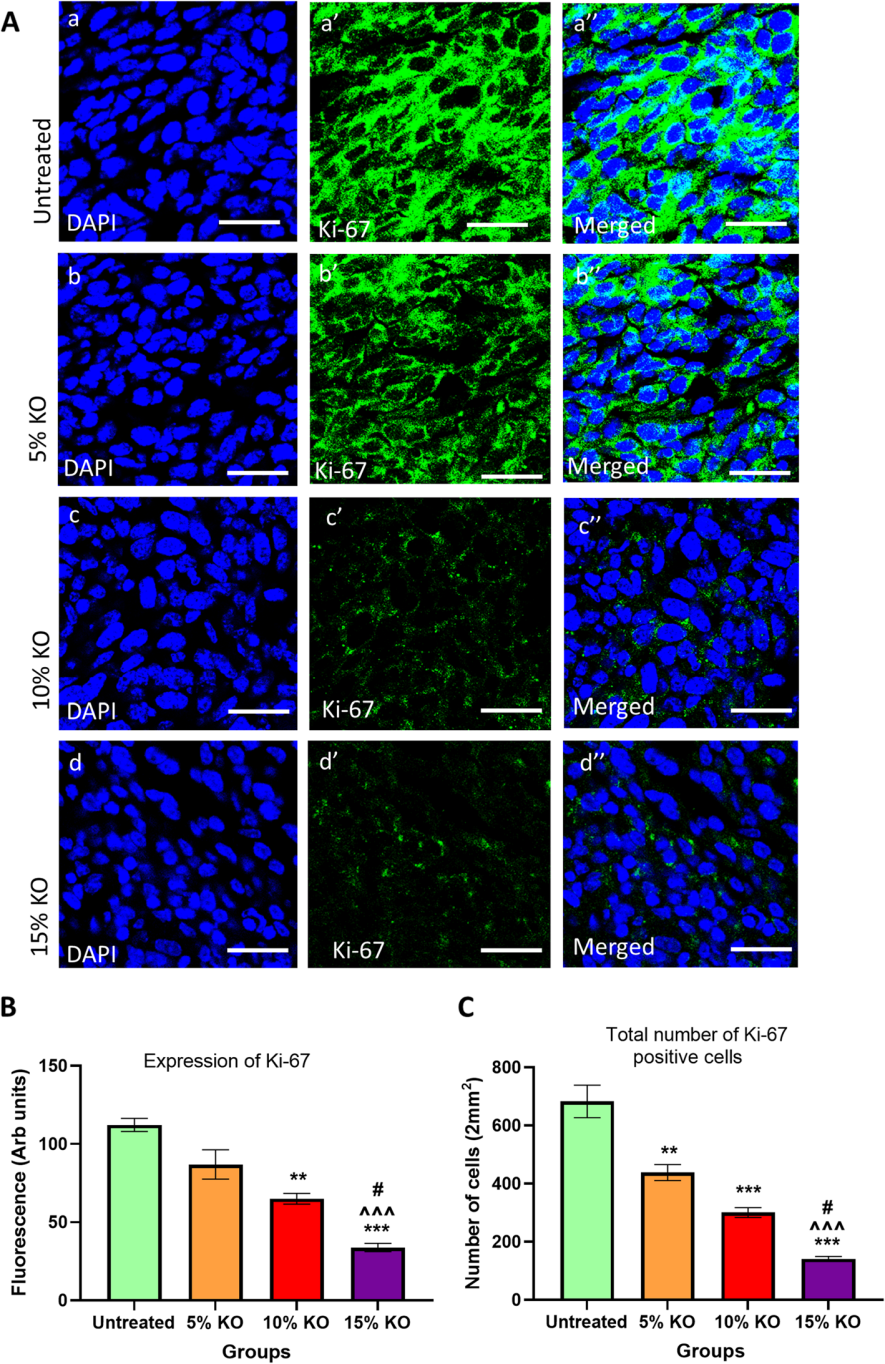
Expression of Ki-67 following treatments with different doses of krill oil. **A** The expression of Ki-67 in tumours following in vivo treatments with different doses of krill oil compared to the untreated group was determined using a monoclonal antibody for Ki-67. **B** The fluorescence intensity of Ki-67 expression in tumours following treatments with different doses of krill oil compared to the untreated group. **C** Quantification of Ki-67-positive cells based on 8 images per preparation at 20 × magnification within a total area of 2mm^2^. The thickness of tumour cross-sections = 10 µM. *N* = 5 mice per group. The results are expressed as mean ± SEM, **P < 0.01, and ***P < 0.001 compared to the untreated group; ^^^*P* < 0.001 compared to 5% krill oil-treated group; ^#^*P* < 0.05 compared to 10% krill oil-treated group

Tumour cell proliferation was analysed immunohistochemically using a proliferation marker Ki-67 (Fig. 4A). The animals fed the diets supplemented with 5% and 10% of krill oil showed a reduction of the number of proliferative cells in the tumour by 22.5 ± 5.2% and 42.0 ± 2.6% (*P* < 0.01 and *P* < 0.001), respectively, compared to the untreated group. The animals fed with 15% of krill oil showed the lowest number of Ki-67 positive cells in the tumour, with a significant reduction by 69.9 ± 2.0% (*P* < 0.001) compared to the untreated group. This is significantly lower compared to animals fed with 10% of krill oil by 25.2% (*P* < 0.05) and animals fed with 5% of krill oil by 61.1% (*P* < 0.001) (Figs. 4B and C).

### Effects of different doses of krill oil supplementation on tumour vascularisation

**Fig. 5:**
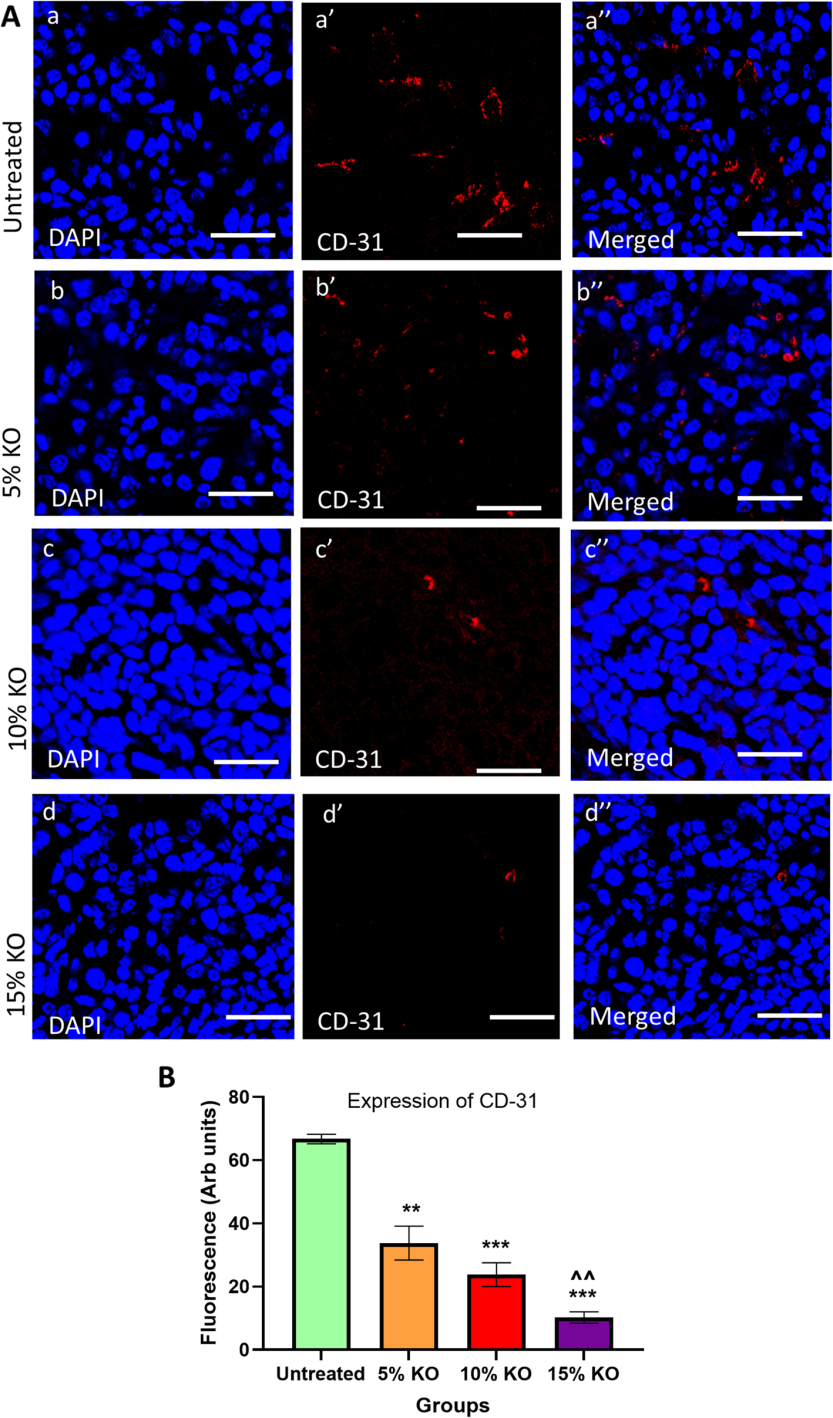
Expression of CD-31 following treatments with different doses of krill oil. (**A**) The expression of CD-31 in tumours following in vivo treatments with different doses of krill oil compared to the untreated group was determined using a monoclonal antibody for CD-31. (**B**) The fluorescent intensity of CD-31 expression in tumours following treatments with different doses of krill oil compared to the untreated group. Thickens of tumour cross-sections = 10 µM. *N* = 5 mice per group, the results are expressed as mean ± SEM, **P < 0.01, and ***P < 0.001 compared to the untreated group; ^^*P* < 0.01 compared to the 5% krill oil-treated group

The tumour vascularisation was determined using the endothelial cell marker CD-31 (Fig. 5A). The animals received diets with krill oil supplementations at concentrations of 5%, 10%, and 15% have shown a significantly lower expression of CD-31 by 49.3% (*P* < 0.01), 64.3% (*P* < 0.001), and 72.7% (*P* < 0.001) respectively compared to untreated animals. Moreover, CD-31 expression in tumours from mice treated with 15% of krill oil was significantly lower compared to mice treated with 5% of krill oil (*P* < 0.01). This indicates that krill oil supplementation inhibits the blood vessel formation in colorectal tumours in a dose-dependent manner (Fig. 5B).

### Expression of pEGFR/EGFR, pERK/ERK 1/2 and pAKT/AKT following krill oil supplementation

**Fig. 6:**
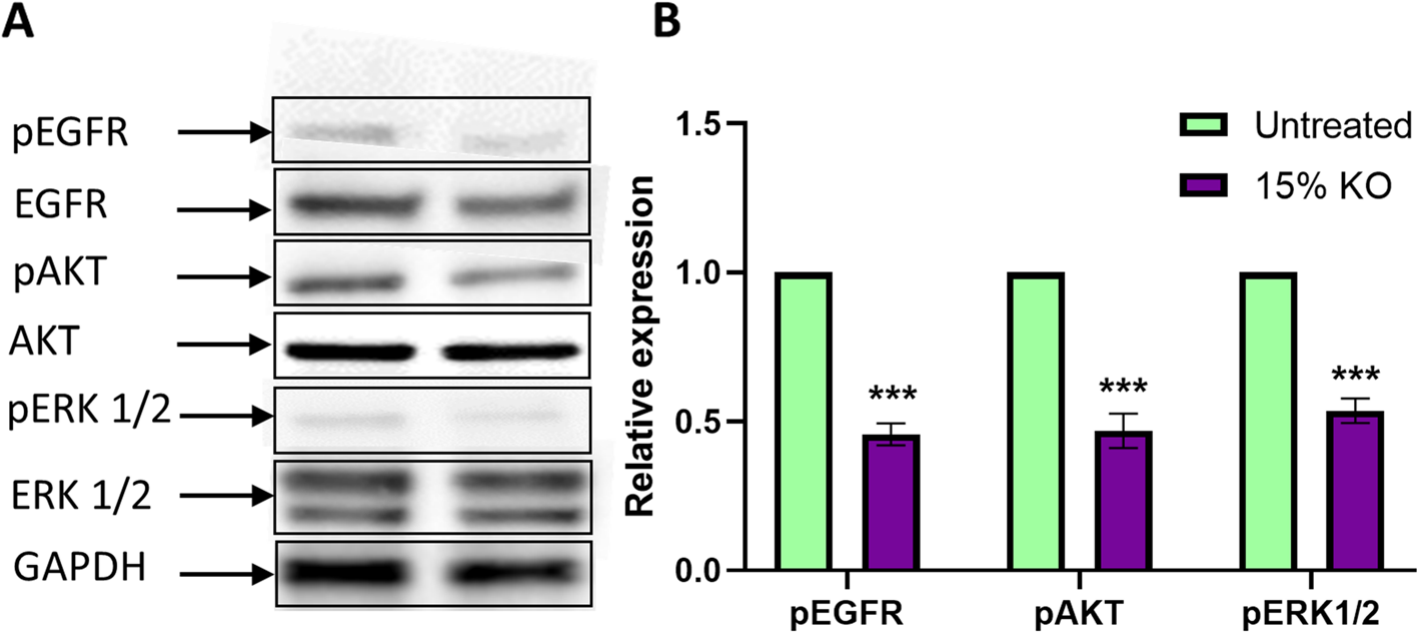
Expression of pEGFR, EGFR, pAKT, AKT pERK ½, and ERK 1/2 following treatments with 15% of krill oil. The expression of pEGFR, EGFR, pAKT, AKT, pERK1/2, and ERK1/2 was analysed by western blotting in tumours following treatment with a 15% dietary krill oil supplementation compared to the untreated group **A**. The relative levels of pEGFR, pAKT, and pERK1/2 expression in tumour tissues after treatment with 15% krill oil compared to tumour tissues from untreated Balb/c mice **B**. *N* = 5 mice per group. The results are expressed as mean ± SEM, ***P < 0.001 compared to the untreated group

The expressions of pEGFR, EGFR, pAKT, AKT, pERK1/2, and ERK1/2 protein levels were investigated in tumours from the animals fed with 15% of krill oil (Fig. 6A). The western blot results revealed that the expression levels of EGFR, AKT, and total ERK1/2 were not changed in the animals fed with 15% of krill oil compared to the untreated animals. However, the expression of pEGFR, pAKT, and pERK1/2were significantly lower in the animals fed with 15% of krill oil with 0.45, 0.54 and 0.46 folds reduction, respectively (*P* < 0.001 for all) compared to the untreated animals (Fig. 6B).

### Expression of cleaved caspase-7, PARP, and DNA/RNA damage following krill oil supplementation

**Fig. 7:**
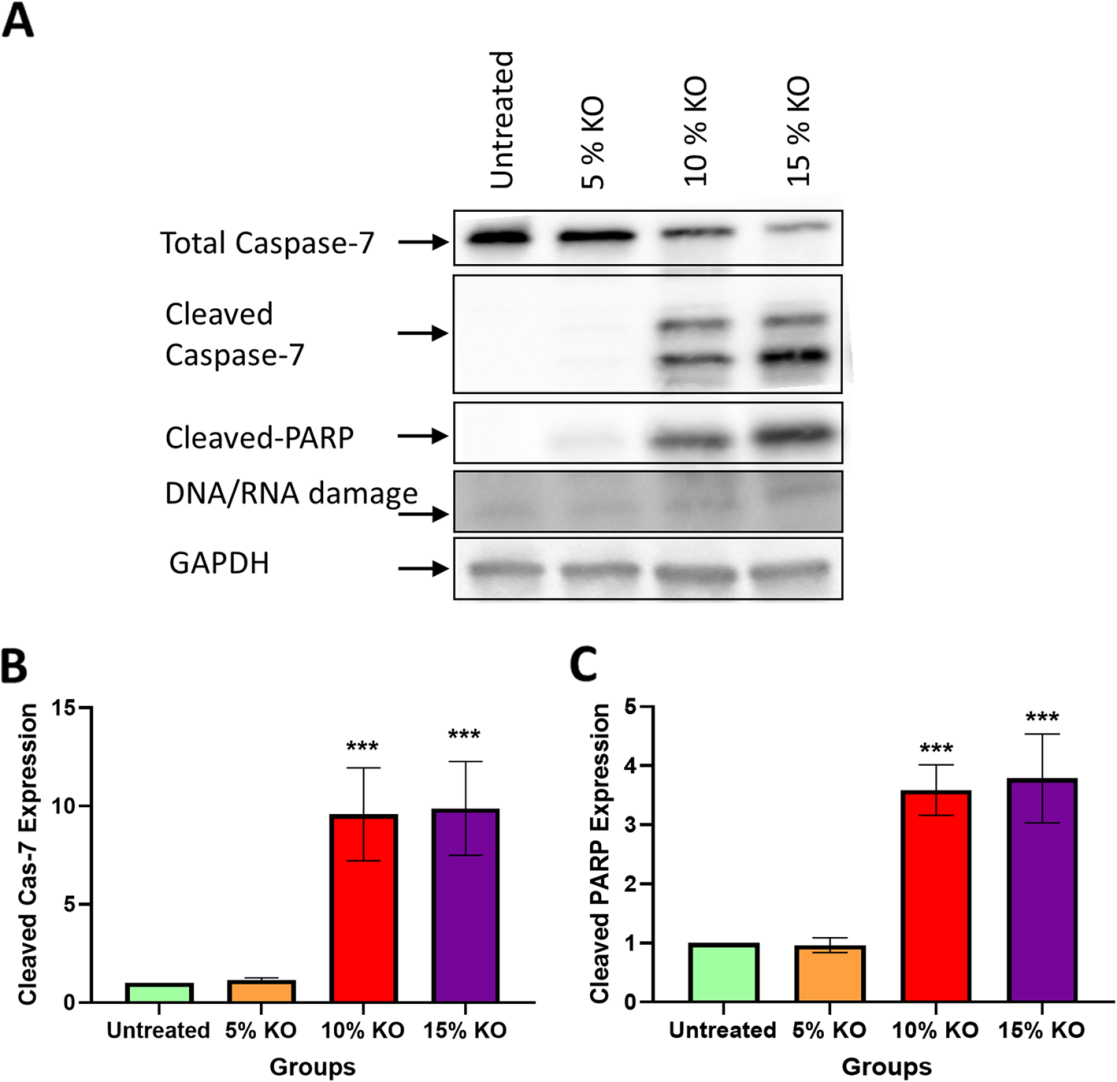
Expression of caspase-7, cleaved caspase-7, cleaved PARP, and DNA/RNA damage following treatments with different doses of krill oil. The expression of Caspase-7, cleaved caspase-7, cleaved PARP, and DNA/RNA damage was analysed by western blotting in tumours following treatment with different doses of krill oil compared to the untreated group **A**. The relative level of cleaved caspase-7 expression in tumours following treatment with different doses of krill oil compared to tumour tissues from untreated Balb/c mice **B**. The relative level of cleaved PARP expression in tumours after treatment with different doses of krill oil compared to tumour tissues from untreated Balb/c mice **C**. *N* = 5 mice per group. The results are expressed as mean ± SEM, ***P < 0.001 compared to the untreated group

The results of western blot analysis of tumours showed that krill oil supplementation activates caspase-7 in a dose-dependent manner (Fig. 7A). The animals fed diets supplemented with 10% and 15% of krill oil showed an increase in the expression of cleaved caspase-7 by 9.6 and 9.9 folds, respectively, compared to the untreated animals (*P* < 0.001). The difference between the group fed with 5% of krill oil and the untreated group was not statistically significant (Fig. 7B).

Furthermore, the animals fed with 10% and 15% of krill oil showed a dose-dependent change in the expression of PARP protein with an increase of 3.6 and 3.9 folds, respectively, compared to the untreated animals (*P* < 0.001 for both) (Fig. 7C). Moreover, the increase in the level of DNA/RNA damage was found following the treatments with 10% and 15% of krill oil by 31.8% and 34.2%,  respectively, compared to untreated animals (Fig. 7A).

## Discussion

In this study, we have demonstrated that dietary krill oil supplementation inhibits the growth of CRC tumour in Balb/c mice. The main findings of the study include: 1) krill oil supplementation reduces tumour volume, invasion, and metastasis in a dose-dependent manner within a range of 10–15% of daily feed intake, and 15% of krill oil being the most effective dose; 2) krill oil supplementation correspondingly reduces tumour cell proliferation and angiogenesis; 3) krill oil downregulates the expression of pEGFR, and its downstream pERK1/2 and pAKT signalling pathways in colorectal tumours in the same fashion as reported in our previous in vitro study using the free fatty acid extract of krill oil; 4) krill oil supplementation upregulates the expression of cleaved caspase-7, cleaved PARP, and DNA/RNA damage in tumours.

The hallmarks of cancer include uncontrolled cell proliferation, inhibition of apoptosis and induction of angiogenesis, resulting in the growth and progression of the solid tumour [29]. Disruption of the tightly regulated EGFR pathway was found to be involved in many cellular processes, including proliferation, growth, and survival of neoplastic cells [30, 31]. Many studies have observed the activation of various carcinogenic events that induce cancer development and progression. Therefore, targeting a single event is not sufficient for tumour control [29, 32]. Accordingly, most cancer treatments aim to control multiple events rather than a single factor in order to suppress CRC progression [33, 34], such as inhibiting cell survival, signalling pathways, and angiogenesis, as well as inducing cellular apoptosis [35, 36]. Hence, in the present study, we investigated the anti-tumour potential of krill oil in a pre-clinical animal model of CRC, by examining its impact on various molecular markers/signalling pathways that are related to cell proliferation, invasion, angiogenesis, and apoptosis.

Krill oil contains a higher level of LC n-3 PUFA, EPA and DHA, and the anti-cancer properties of these LC n-3 PUFA have been reported in both in vitro [37, 38] and in vivo studies [25, 39]. For example, Morin et al. [24] have evaluated the anti-cancer effect of EPA monoglyceride (MAG-EPA) and krill oil treatment on HCT-116 cells and also in a mouse model of HCT-116 xenografts. They found that MAG-EPA at the dose of 618 mg/kg/day reduced cell proliferation and tumour size by up to 75%, but no significant reduction was observed after krill oil treatment in vivo. In their study, the administrated dose of krill oil was 618 mg/kg/day (less than 5%). The current study also showed that treatment with 5% of krill oil does not result in positive effects against CRC. The study by Latham et al. [40] observed that fish oil at the dose of 80 g/kg (EPA 18.7% and DHA 8.0%) consumption significantly reduces the incidents of aberrant crypt foci (ACF) in the distal colon following administration of 1,2-dimethylhydrazine (DMH) to induce cancer in Wistar rats. Rosa et al. [41] also reported that 4% of fish oil-supplemented diet given to Wister rats resulted in a significant reduction of ACF formation in the proximal colon after receiving DMH injection.

Our study showed that 15% of krill oil supplementation reduces tumour weight and volume by 68.5% and 68.3%, respectively, compared to untreated animals. The inhibitory effects of krill oil on tumour growth are most likely associated with the suppression of proliferation and induction of apoptosis of cancer cells. This was supported by our results demonstrating that krill oil supplementation induces apoptosis and downregulation of Ki-67 in the tumours. The Ki-67 is a biomarker for the proliferation of tumour cells and is a regulatory protein associated with the nuclear cell cycle. Its expression can be detected during the interface in the nucleus of tumour epithelial cells [42, 43]. In addition, Ki-67 is involved in the entire active phases of the cell cycle, G1, S, G2, and mitosis, but is absent in the resting phase of the cell cycle (G0 phase) [44]. Therefore, Ki-67 is an excellent marker for determining the growth fraction of tumours [45]. These in vivo results are consistent with our previous findings demonstrating that the FFAE of krill oil exhibited a significant anti-proliferative effect on human osteosarcoma and CRC cells [20, 22].

The formation of a vascular network is crucial for increased cell proliferation and metastatic spread of tumour as this network supplies oxygen, nutrients, and eliminates waste products that are essential for the progression of cancer [46, 47]. Furthermore, tumour-associated angiogenesis is believed to be dependent on the production of pro-angiogenic growth factors by tumour cells. These factors enhance tumour angiogenesis to promote tumour growth [48]. Studies also showed that suppression of angiogenesis obstructs tumour growth and progression [49]. Therefore, therapeutic approaches that can inhibit angiogenesis would help to control tumorigenesis [50, 51]. In the present study, we found that higher doses of krill oil (10–15%) significantly inhibit blood vessel formation in colorectal tumours, evident by the reduced expression of platelet endothelial cell adhesion molecule CD-31. CD-31 is highly expressed on the surface of all vascular cells. It is a well-defined marker for identifying angiogenesis and also well-established for monitoring blood vessel density in malignant tissues [52, 53]. On the contrary, a higher expression of this biomarker was observed in the untreated group with highly aggressive tumours. There was no significant change in the expression of CD-31 following treatment with the low dose of krill oil (5%) compared to the untreated group. The effect of krill oil on CD-31 may be attributed to the presence of LC n-3 PUFA in this marine oil. Previously, Hawcroft et al. [54] have demonstrated low doses of EPA-FFA at 2.5 to 5% ww-1 did not decrease the density of CD31-positive microvessels in CRC tumour tissues which correlated with the lack of CRC cell apoptosis in metastatic liver tumours in Balb/c mice. These findings are consistent with the results of our study, demonstrating that only higher doses of krill oil supplementation (10–15%) were effective in reducing CD-31 expression, but not the lower doses of krill oil (5%). A decreased density of CD-31- positive microvessels in Met-1 tumours from FVB female mice was observed following 17 days of treatment with epoxydocosapentaenoic acids (EDPs), lipid mediators produced from DHA by cytochrome P450 epoxygenases [55].

In this study, we observed the downregulation of phosphorylated EGFR and its ERK1/2 and AKT downstream signalling pathways with no alternations of their total protein levels in tumours following krill oil supplementation in vivo. These results are consistent with our previous findings that the free fatty acid extract of krill oil inhibits the expression of EGFR and its downstream signalling pathway, leading to CRC cancer cell death [56]. EGFR signalling pathways are responsible for various biological processes, including cell growth, proliferation, and survival [57]. The overexpression of EGFR and its ERK1/2 and AKT downstream signalling pathways is common in cancer including CRC. The EGFR mutation results in dysregulation of several biological processes, including cell proliferation, apoptosis, and survival [58, 59]. Furthermore, previous studies have observed that activation of the EGFR pathway also increases the synthesis of angiogenic molecules in different types of tumour cells [60–63]. Taken together, it suggests that, the anti-cancer properties of krill oil observed in the present study may be associated with the downregulation of pEGFR, pERK1/2, and pAKT signalling, leading to the inhibition of tumour cell proliferation, angiogenesis and apoptosis.

Furthermore, the anti-proliferative and pro-apoptotic effects of krill oil may be attributed to its role in the activation of caspase-7, hence inducing cellular apoptosis through the cleavage of PARP and finally induced DNA/RNA damage. The extrinsic and intrinsic apoptotic signalling cascades are the two commonly distinguished independent apoptotic pathways [64]. The interaction of death receptor ligands causes the activation of the extrinsic apoptotic pathway through activation of caspase-8 and caspase-10. On the other hand, the intrinsic apoptotic pathway is triggered by the change of mitochondrial membrane potential and release of cytochrome *c* in the cytosol and this causes the formation of the apoptosome complex to combine with caspase-9. Once activated, caspase-9 leads to the activation of the executioner caspase-3 and -7 to induce apoptosis [65, 66]. In our previous in vitro study, we have reported the role of FFAE of krill oil in the activation of caspase-9 and -3. The results of the present in vivo study demonstrate almost tenfold increases in the expression of cleaved caspase-7 in tumours from mice with 10% and 15% krill oil supplementation. It has been reported that there is a functional link between caspase-7 and PARP cleavage involved in both inflammation and apoptotic processes [67]. While the current study has demonstrated that krill oil supplementation reduces CRC tumour growth via the activation of caspase-7, which results in PARP cleavage to induce DNA damage, Lamkanfi et al. [67] have reported that the caspase-7-induced apoptosis depends on the cell type and stimulus type. Further studies are required to elucidate whether krill oil supplementation affects inflammatory pathways leading to caspase-7 activation.

Collectively, the present study shows that krill oil supplementation inhibits colorectal tumours growth in Balb/c mice. This effect is most likely related to the anti-proliferative, anti-angiogenic, and pro-apoptotic properties of krill oil. In addition, krill oil supplementation did not show any side effects, such as vomiting, diarrhoea, or constipation throughout the study period. There were also no adverse effects on food intake and body weight. This is consistent with the previous studies on SD rats and Wistar rats. Robertson et al. [68] have found that 13-week supplementation with krill oil in Wistar rats has not caused adverse toxicological effects on animals. The studies by Zhu et al. [21] and Ramsvik et al. [69] observed that treatment with krill oil can control the serum lipid level without any side effects.

The limitation of this pilot study is that only a small number of animals were used for each treatment. An increase in the number of animals in each group would improve the statistical power. Furthermore, the study did not include treatments with isolated active components of krill oil, including EPA, DHA, and astaxanthin, therefore it is not possible to identify the specific roles of these components in relation to the anti-cancer properties of krill oil. In addition, the activity and RT-PCR of Caspase 7 were not measured in this study. Future investigation on these aspects will help to further understand the molecular mechanisms underlying the anti-tumour effects of krill oil.

## Conclusion

This pilot animal study has demonstrated that krill oil supplementation reduces CRC tumour growth in a dose-dependent manner (5% to15%). The anti-tumour effects of krill oil may be associated with the suppression of CRC cell proliferation through the downregulation of EGFR signalling pathways in the same manner as in our previous in vitro study as well as the activation of caspase-7 to induce apoptosis and inhibition of tumour angiogenesis. The results of this pilot study could be used as a basis for larger animal studies or human clinical trials to further explore the anti-cancer potential of krill oil.

## Supplementary Information

**Additional file 1.** Supplementary Figure 6 A. Supplementary Figure 7.

## Data Availability

The datasets from the present study are available from the corresponding author upon request.

## Abbreviations

AKT: Protein kinase B
ATCC: American tissue culture collection
CD-31: Platelet endothelial cell adhesion molecule
CRC: Colorectal cancer
DAPI: 4,6-Diamidino-2-phenylindole
DHA: Docosahexaenoic acid
EGFR: Epidermal growth factor receptor
EPA: Eicosapentaenoic acid
ERK: Extracellular signal regulated kinase
FCS: Foetal calf serum
FFA: Free fatty acids
FFAE of KO: Free fatty acids extract of krill oil
GAPDH: Glyceraldehydes-3-phosphate de-hydrogenase
HEPES: 4-(2-Hydroxyethyl)-1-piperazineethanesulfonic acid
LC n-3 PUFA: Long chain n-3 polyunsaturated fatty acids
MAPK: Mitogen-activated protein kinase
OCT: Optimal cutting temperature compound
pAKT: Phosphorylated AKT
PBS: Phosphate-buffered saline
PBS-T: Phosphate-buffered saline + Tween 20
pEGFR: Phosphorylated EGFR
pERK: phosphorylated ERK
PARP: Cleaved poly (ADP-ribose) polymerase
PUFA: Polyunsaturated fatty acids
RIPA: Radioimmunoprecipitation assay buffer
RPM: Revolutions per minute
SD: Standard deviation
SDS: Sodium dodecyl sulphate
SEM: Standard error of mean
TP53: Tumour protein p53
