# Supplementary material for: Krill oil supplementation reduces the growth of CT-26 orthotopic tumours in Balb/c mice
Source: BMC Complement Med Ther. 2022 Feb 4;22:34. doi: 10.1186/s12906-022-03521-4 (PMC8817584; doi:10.1186/s12906-022-03521-4)
Supplement: Supplementary file 1 — Additional file 1. Supplementary Figure 6 A. Supplementary Figure 7. [file 12906_2022_3521_MOESM1_ESM.pptx]

## Slide 1
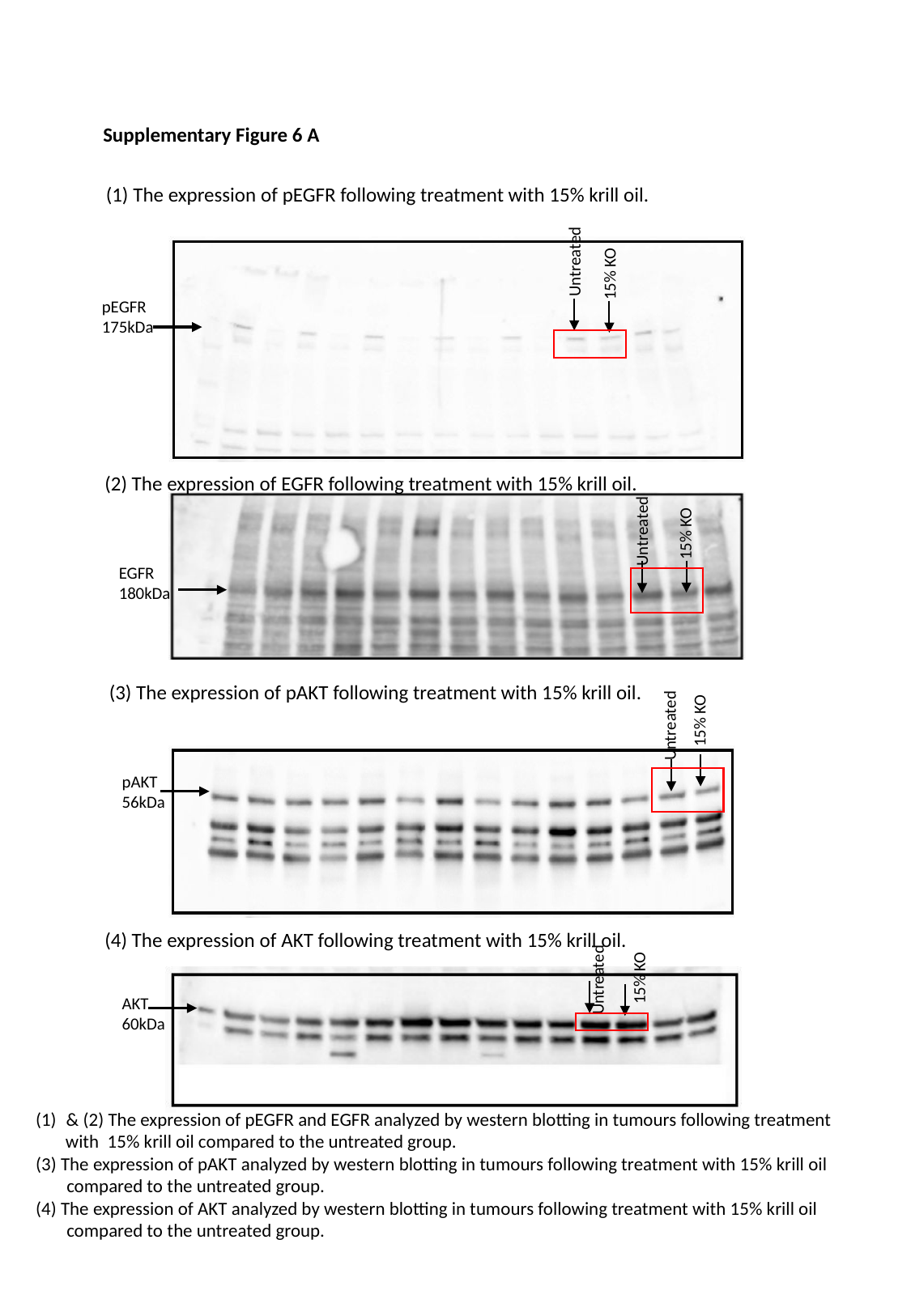

Supplementary Figure 6 A
(1) The expression of pEGFR following treatment with 15% krill oil.
Untreated
15% KO
pEGFR
175kDa
15% KO
Untreated
EGFR
180kDa
(2) The expression of EGFR following treatment with 15% krill oil.
15% KO
Untreated
pAKT
56kDa
(3) The expression of pAKT following treatment with 15% krill oil.
(4) The expression of AKT following treatment with 15% krill oil.
15% KO
Untreated
AKT
60kDa
& (2) The expression of pEGFR and EGFR analyzed by western blotting in tumours following treatment
 with 15% krill oil compared to the untreated group.
(3) The expression of pAKT analyzed by western blotting in tumours following treatment with 15% krill oil compared to the untreated group.
(4) The expression of AKT analyzed by western blotting in tumours following treatment with 15% krill oil compared to the untreated group.

## Slide 2
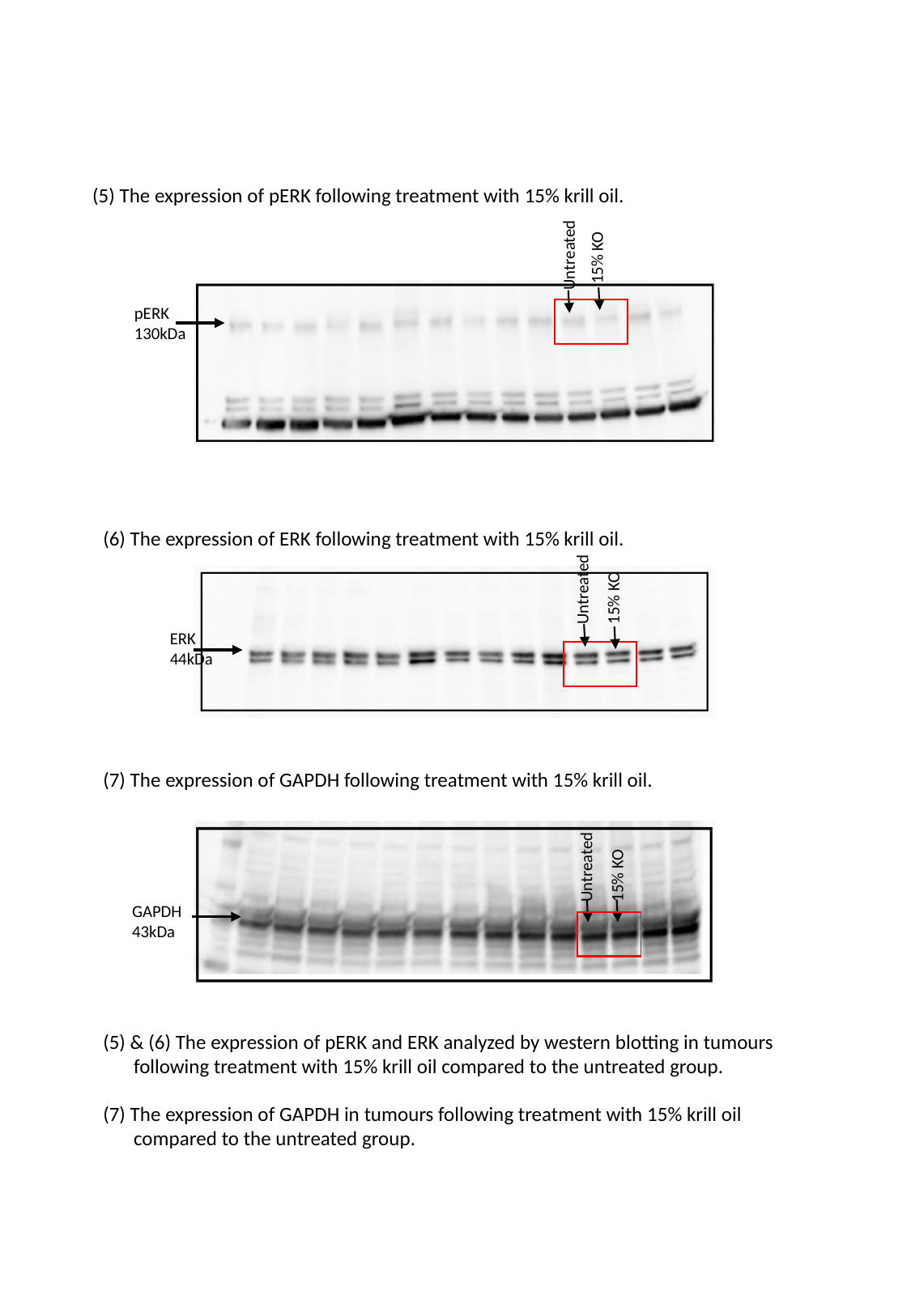

(5) The expression of pERK following treatment with 15% krill oil.
15% KO
Untreated
pERK
130kDa
15% KO
Untreated
ERK
44kDa
(6) The expression of ERK following treatment with 15% krill oil.
(7) The expression of GAPDH following treatment with 15% krill oil.
15% KO
Untreated
GAPDH
43kDa
(5) & (6) The expression of pERK and ERK analyzed by western blotting in tumours following treatment with 15% krill oil compared to the untreated group.
(7) The expression of GAPDH in tumours following treatment with 15% krill oil compared to the untreated group.

## Slide 3
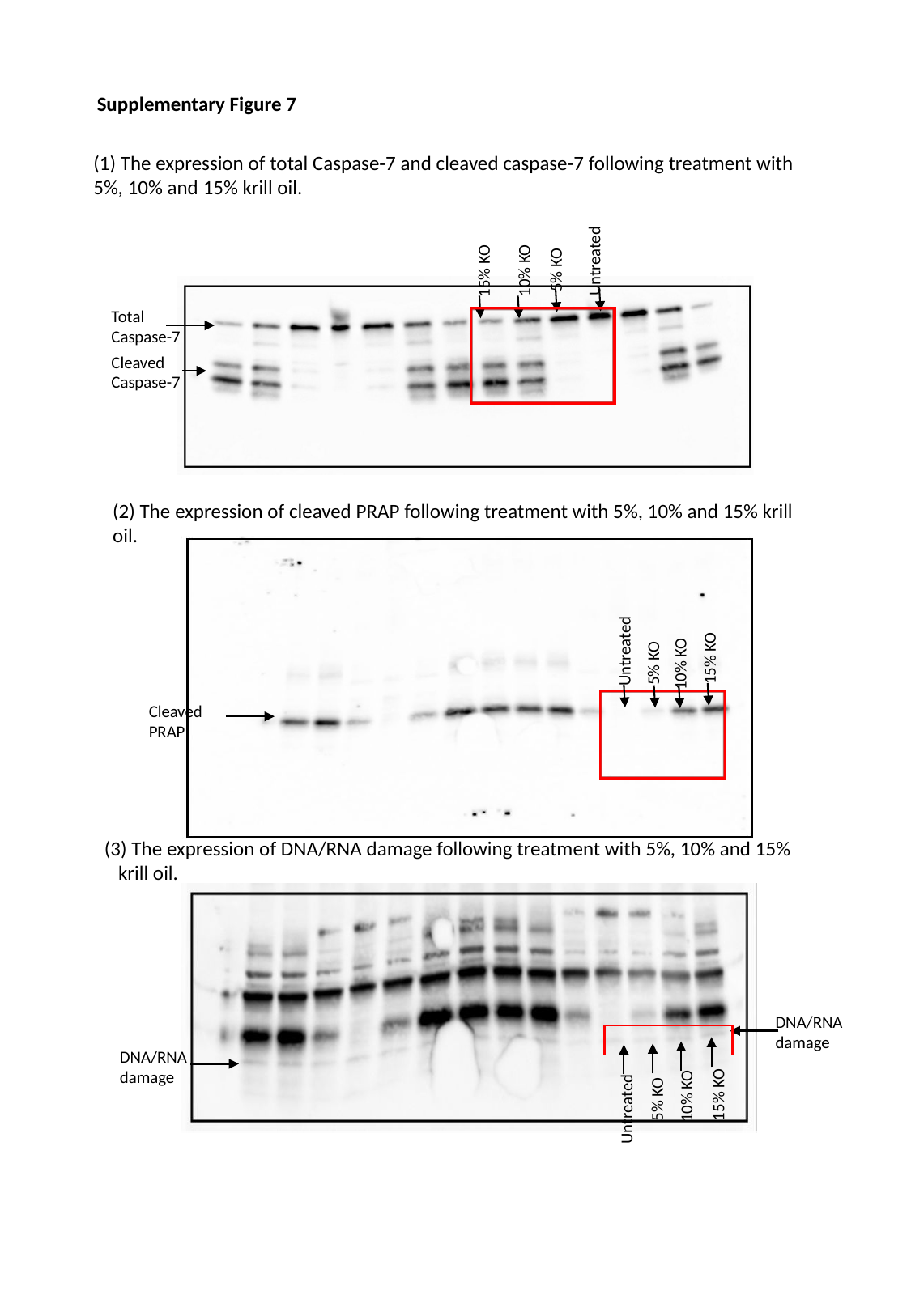

Supplementary Figure 7
(1) The expression of total Caspase-7 and cleaved caspase-7 following treatment with 5%, 10% and 15% krill oil.
5% KO
Untreated
10% KO
15% KO
Total Caspase-7
Cleaved Caspase-7
(2) The expression of cleaved PRAP following treatment with 5%, 10% and 15% krill oil.
15% KO
5% KO
Untreated
10% KO
Cleaved
PRAP
(3) The expression of DNA/RNA damage following treatment with 5%, 10% and 15% krill oil.
DNA/RNA damage
DNA/RNA damage
5% KO
15% KO
10% KO
Untreated

## Slide 4
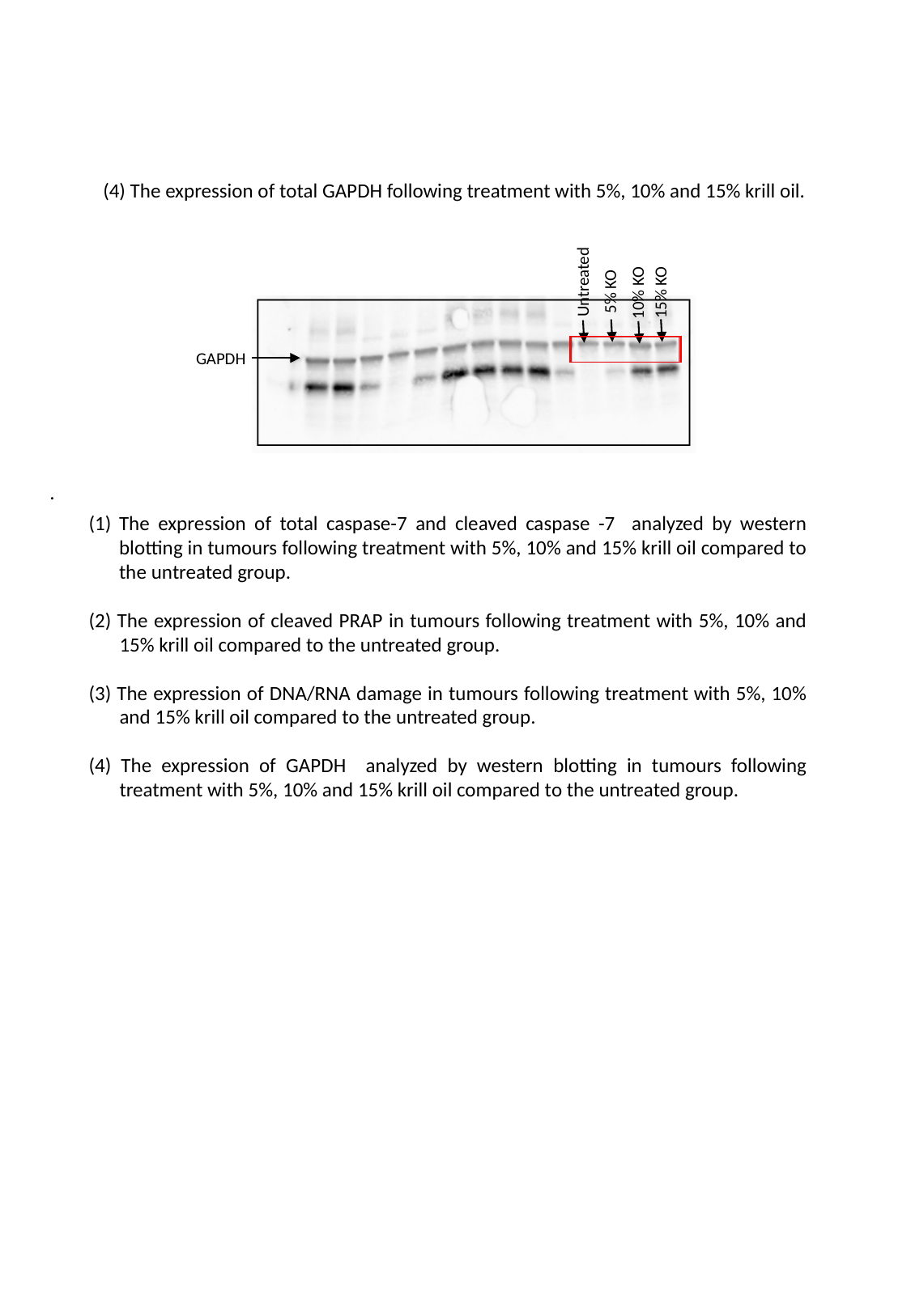

(4) The expression of total GAPDH following treatment with 5%, 10% and 15% krill oil.
5% KO
Untreated
15% KO
10% KO
GAPDH
.
The expression of total caspase-7 and cleaved caspase -7 analyzed by western blotting in tumours following treatment with 5%, 10% and 15% krill oil compared to the untreated group.
(2) The expression of cleaved PRAP in tumours following treatment with 5%, 10% and 15% krill oil compared to the untreated group.
(3) The expression of DNA/RNA damage in tumours following treatment with 5%, 10% and 15% krill oil compared to the untreated group.
(4) The expression of GAPDH analyzed by western blotting in tumours following treatment with 5%, 10% and 15% krill oil compared to the untreated group.
